# Genetic Heterogeneity of Susceptibility Gene in Different Ethnic Populations: Refining Association Study of *PTPN22* for Graves’ Disease in a Chinese Han Population

**DOI:** 10.1371/journal.pone.0084514

**Published:** 2013-12-30

**Authors:** Liqiong Xue, Chunming Pan, Zhaohui Gu, Shuangxia Zhao, Bing Han, Wei Liu, Shaoying Yang, Shasha Yu, Yixuan Sun, Jun Liang, Guanqi Gao, Xiaomei Zhang, Guoyue Yuan, Changgui Li, Wenhua Du, Gang Chen, Jialun Chen, Huaidong Song

**Affiliations:** 1 State Key Laboratory of Medical Genomics, Ruijin Hospital Affiliated to Shanghai Jiaotong University (SJTU) School of Medicine, Shanghai, China; 2 Shanghai Center for Systems Biomedicine, SJTU, Shanghai, China; 3 Shanghai Institute of Endocrinology and Metabolism, Ruijin Hospital Affiliated to SJTU School of Medicine, Shanghai, China; 4 Department of Endocrinology, Shanghai Ninth People’s Hospital, Shanghai Jiao Tong University School of Medicine, Shanghai, China; 5 Department of Geriatric Medicine, East Hospital Affiliated to Tongji University School of Medicine, Shanghai, China; 6 Department of Endocrinology, the Central Hospital of Xuzhou Affiliated to Xuzhou Medical College, Xuzhou, Jiangsu Province, China; 7 Department of Endocrinology, Linyi People’s Hospital, Linyi, Shandong Province, China; 8 Department of Endocrinology, the First Hospital Affiliated to Bengbu Medical College, Bengbu, Anhui Province, China; 9 Department of Endocrinology, the Hospital Affiliated to Jiangsu University, Zhenjiang, Jiangsu Province, China; 10 Department of Endocrinology and Gout Laboratory, Medical School Hospital of Qingdao University, Qingdao, Shandong Province, China; 11 Department of Endocrinology, Fujian Province Hospital, Fuzhou, Fujian Province, China; Kunming Institute of Zoology, Chinese Academy of Sciences, China

## Abstract

In our previous studies, we presumed subtypes of Graves’ disease (GD) may be caused by different major susceptibility genes or different variants of a single susceptibility gene. However, more evidence is needed to support this hypothesis. Single-nucleotide polymorphism (SNP) rs2476601 in *PTPN22* is the susceptibility loci of GD in the European population. However, this polymorphism has not been found in Asian populations. Here, we investigate whether *PTPN22* is the susceptibility gene for GD in Chinese population and further determine the susceptibility variant of *PTPN22* in GD. We conducted an imputation analysis based on the results of our genome-wide association study (GWAS) in 1,536 GD patients and 1,516 control subjects. Imputation revealed that 255 common SNPs on a linkage disequilibrium (LD) block containing *PTPN22* were associated with GD (*P*<0.05). Nine tagSNPs that captured the 255 common variants were selected to be further genotyped in a large cohort including 4,368 GD patients and 4,350 matched controls. There was no significant difference between the nine tagSNPs (*P*>0.05) in either the genotype distribution or allelic frequencies between patients and controls in the replication study. Although the combined analysis exhibited a weak association signal (*P*_combined_ = 0.003263 for rs3811021), the false positive report probability (FPRP) analysis indicated it was most likely a false positive finding. Our study did not support an association of common SNPs in *PTPN22* LD block with GD in Chinese Han population. This suggests that GD in different ethnic population is probably caused by distinct susceptibility genes.

## Introduction

Graves’ disease (GD) is one of the most common autoimmune diseases (AIDs) and is characterized by the production of autoantibodies that bind and stimulate the thyroid-stimulating hormone receptor (TSHR), resulting in hyperthyroidism and diffuse enlargement of the thyroid gland. GD is universally considered to be a complex disease triggered by the interaction between susceptibility genes [1-3] and non-genetic factors, such as stress, iodine intake, and infection[4,5]. The prevalence of GD is approximately 0.5–2% in Western countries and 2–3.0% in China[6,7]. Family and twin studies showing that 79% of the predisposition to the development of GD is attributable to genetic factors[8], thereby it is of importance to identify the susceptibility genes and loci, which will facilitate diagnosis, prevention, and treatment of this disease.

Seven susceptibility loci, including human leukocyte antigen (HLA), cytotoxic T lymphocyte antigen 4 (*CTLA-4*), Fc receptor-like 3 (*FCRL3*), ribonuclease T2 (*RNASET2*), secretoglobin, family 3A member 2 (*SCGB3A2*), thyroid-stimulating hormone receptor (TSHR), and thyroglobulin (TG), have been widely confirmed to be associated with GD in different ethnic populations[9-20]. In addition, a gene named protein-tyrosine-phosphate nonreceptor 22 (*PTPN22*) has previously been reported as a susceptibility locus for Graves’ disease (GD)[21,22] in European populations. However, its role in GD predisposition in Asian populations is still controversial[23].

*PTPN22* is located at chromosome 1p13.2 and encodes the intracellular tyrosine phosphatase LYP, which acts as a negative regulator in early T-cell activation and signal transduction through binding to the Csk protein[24]. A functional single-nucleotide polymorphism (SNP) R620W (rs2476601) at position +1858 (+1858C/T) was first identified as a susceptibility locus to Type 1 diabetes (T1D) in a European population[25]. The variant was further reported to be associated with several AIDs, such as Rheumatoid arthritis (RA), autoimmune thyroid disease, and systemic lupus erythematosus[21,25-27]. However, it is noteworthy that the polymorphism of rs2476601 was reported monomorphic in Asian populations[19,28-30], which indicates that it may not have a causal role for GD in the Asian population.

In our previous studies, we presumed that subtypes of GD may be caused by different major susceptibility genes or different variants of a single susceptibility gene[12,17]. Given the genetic heterogeneity of *PTPN22* in different ethnic populations, we intend to investigate the association of SNPs in *PTPN22* with GD in a large number of samples in order to define whether *PTPN22* is the susceptibility gene of GD in Chinese Han population.

## Materials and Methods

### Subjects and sample collection

We enrolled 5,904 GD patients (4,635 females and 1,269 males; age 39 ±14 yr) and 5,866 geographically matched healthy controls (4,506 females and 1,360 males; age 48 ± 12 yr) from the Chinese Han population. GD was diagnosed as previously reported[12,16,17]. The patients and control subjects gave their written informed consent, and the project was approved by the local Research Ethics Committee from Ruijin Hospital, the Central Hospital of Xuzhou, the first affiliated hospital of Bengbu Medical College, Medical School Hospital of Qingdao University, Linyi People’s Hospital, the Hospital Affiliated to Jiangsu University, and Fujian Province Hospital respectively. Genomic DNA was extracted from peripheral blood leukocytes using FUJIFILM QuickGene-610L system.

### SNP Selection, Genotyping, and Quality Control (QC) Filters

**Figure 1:**
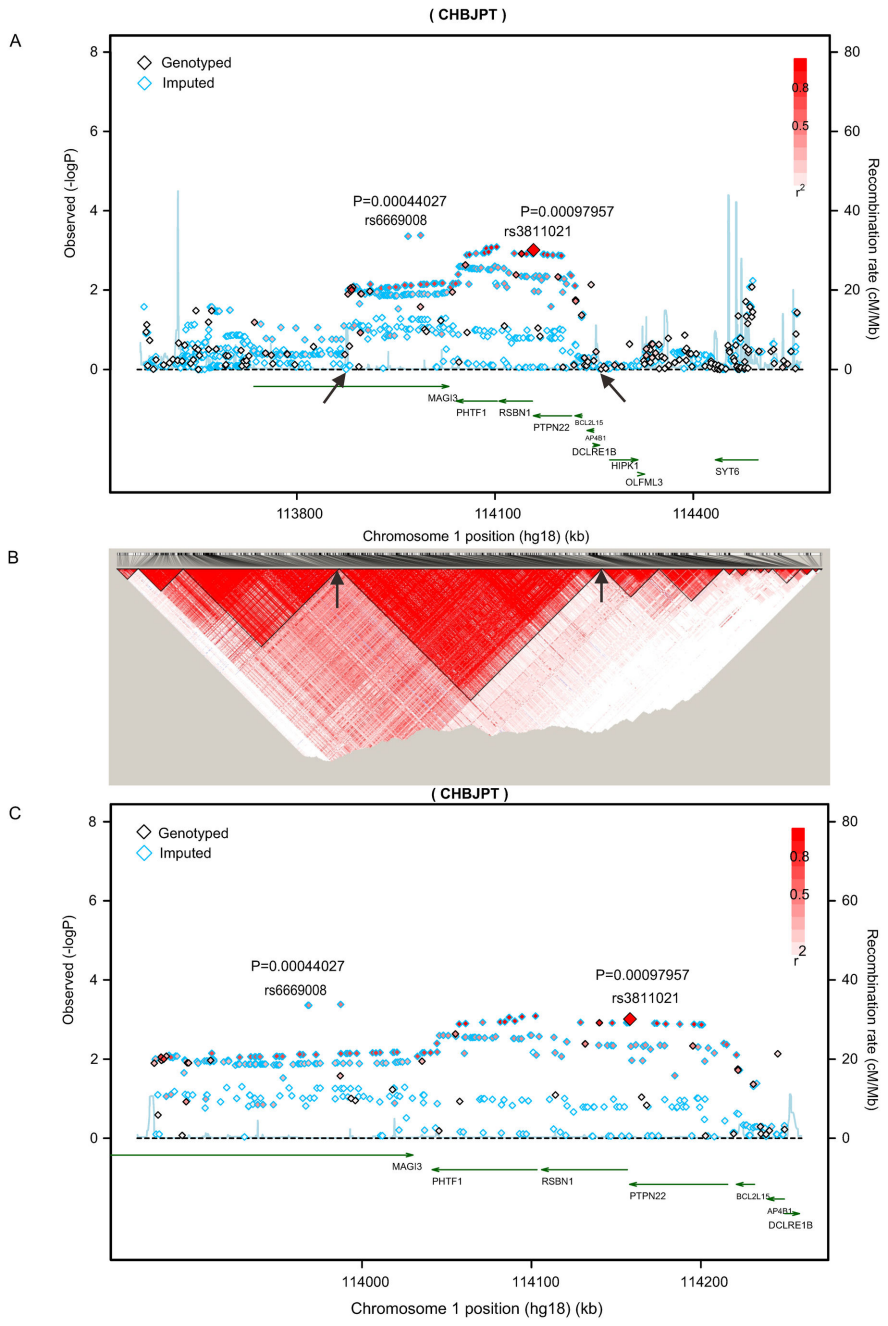
Regional plot of association results in LD block containing *PTPN22* at 1p13.2 in GWAS stage. (A) The results of association for 1,277 genotyped and imputed SNPs in the 1.2-Mb region containing *PTPN22* with Graves’ disease. The color of each SNP spot reflects its r^2^, with the top typed SNP (large red diamond) within each association locus changing from red to white. Genetic recombination rates, estimated using the 1000 Genomes pilot 1 CHB and JPT samples, are shown in cyan. Physical positions are based on NCBI build 36. (B) Linkage disequilibrium plots of the 1,277 SNPs in the 1.2-Mb region containing *PTPN22*. The r^2^ value is estimated by the genotype data of GD cases and controls enrolled in the GWAS. We constructed the plots using Haploview software version 4.2. (C) The plots of the association of 474 genotyped and imputed SNPs with GD. These 474 SNPs are located in a ~445-kb linkage disequilibrium region containing *PTPN22* and were marked with arrows in panels A and B.

DNA samples from 1,536 GD cases and 1,516 controls were genotyped using Illumina Human660-Quad BeadChips at the GWAS stage. Then we performed quality control that excluded call rate< 98%, gender inconsistencies and cryptic relatedness (142 samples). The genotype data for 186 SNPs within large linkage disequilibrium (LD) block region (between 113.7-114.9 MB on chromosome 1, defined by two apparent recombination hotspots) containing *PTPN22* were obtained in a cohort, including 1,442 GD cases and 1,468 controls from our previous GWAS[16]. (Figure 1A)

To further define the loci associated with GD, we performed an imputation analysis based on our GWAS data and obtained the genotype data of 1,277 SNPs in the ~1.2Mb*-*large LD region containing *PTPN22* for subsequent analysis (Figures 1A and 1B; Table S1). Notably, the SNPs associated with GD were mostly located in a small region highlighted by two recombination hotspots (marked by the arrow in Figure 1A), harboring 474 SNPs spanning across a ~370-kb LD block at 1p13.2 around *PTPN22* covering six other known functional genes: *MAGI3, PHTF1, RSBN1, BCL2L15, AP4B1*, and *DCLRE1* (Figures 1A and 1C; and Table S1).

Among the 474 SNPs, 255 SNPs showed a P value less than 0.05, and nine tagSNPs were selected using Haploview software (version 4.2) based on our GWAS and imputation data of 255 SNPs with a criterion of r^2^>0.8 (Table S2). Furthermore, additional 4,368 GD patients and 4,350 matched controls were genotyped for replication using the TaqMan SNP Genotyping Assays, which was a widely accepted method and also referred in our article[20].

The samples with low call rates (<90%) were discarded, and 8,553 samples (4,254 patients and 4,299 controls) were left for further analysis in replication stage. The Hardy-Weinberg disequilibrium for the nine SNPs genotyped in the controls was calculated by Haploview 4.2 software and the *P*-values of the nine tagSNPs were more than 0.05, suggesting that all of the tagSNPs showed no significant deviation from Hardy-Weinberg disequilibrium. 

### Imputation and statistical analysis

We performed the imputation analysis on the *PTPN22-*containing LD region using the program IMPUTE2[23,31,32] together with the observed genotype data and the 1000 Genomes Project phase 1 interim impute data (Jun 2011) as a reference. After imputation and strict quality control (using only SNPs with confidence scores of ≥0.9, call rates ≥95%, and non-deviation from Hardy-Weinberg equilibrium (P >10^-6^ and MAF >1%), our datasets included 1,277 SNPs in the *PTPN22-*containing LD region for subsequent analysis (Table S1).

Association analysis of case-control data at the GWAS stage was conducted by Cochran-Armitage trend test in PLINK^30^. At the replication stage, the association test was assessed using the Cochran-Armitage test for trend applied in PLINK v1.07[30]. Finally, the Cochran-Mantel-Hanezel stratification analysis was used in the combined population. The LD structure was calculated using Haploview version 4.2 software[33]. False positive report probability (FPRP) was analyzed using the FPRP calculation spreadsheet provided by Wacholder et al.[34]

The R package was used to generate the genome-wide *P*-value plot, and the regional plots were generated using SNAP version 2.2 software[35]. 

## Results

### GWAS imputation and association analysis

One hundred eighty-six SNPs spanned across the ~1.2-Mb LD block at 1p13.2 around *PTPN22* and covered 10 other annotated genes: namely, *MAGI3, PHTF1, RSBN1, PTPN22, BCL2L15, DCLRE1, HIPK1, OLFML3, AP4B1*, and *SYT6* (Figure 1A and Table S1). It is worth noting that SNP rs3811021, located in the 3′ UTR of *PTPN22* (downstream of the stop codon of *PTPN22*) exhibited the most significant difference in our genotyped SNPs in the initial GWAS scan (*P*_GWAS_ = 9.80×10^-4^, OR = 1.24; Table 1). However, the previously reported SNP, rs2476601, was monomorphic in our dataset.

**Table 1:** Associaiton results of nine tagSNPs on 1p13.2 region in GD patients and controls.

			GWAS (1,442 vs.1,468)				Replication (4,254 vs. 4,299)				Combined (5,696 vs. 5,767)			
SNP	Chr. Position	Alleles/ genotype	Case frequency	Control frequency	*P* Value	OR (95% CI)	Case frequency	Control frequency	*P* Value	OR (95% CI)	Case frequency	Control frequency	*P* Value	OR (95% CI)
rs7514649	114079822	A<G	0.18*	0.16*	0.0089	1.2 (1.05-1.38)	0.18*	0.17*	0.4332	1.03 (0.95-1.12)	0.18*	0.17*	0.0468	1.07 (1.00-1.15)
		AA	0.03	0.03	0.0305	/	0.03	0.03	0.4620	/	0.03	0.03	0.0805	/
		AG	0.30	0.26			0.29	0.28			0.29	0.28		
		GG	0.67	0.71			0.68	0.69			0.68	0.69		
rs12753075	114127526	T<C	0.09*	0.08*	0.0419	1.21 (1.01-1.45)	0.08*	0.08*	0.7315	0.98 (0.88-1.09)	0.09*	0.08*	0.4635	1.04 (0.94-1.14)
		TT	0.01	0.00	0.1081	/	0.01	0.01	0.9425	/	0.01	0.01	0.7389	/
		TC	0.17	0.15			0.15	0.15			0.02	0.15		
		CC	0.82	0.85			0.84	0.84			0.97	0.84		
rs1217223	114140889	G<A	0.33*	0.29*	0.0133	1.16 (1.03-1.29)	0.31*	0.31*	0.4535	1.03 (0.96-1.09)	0.31*	0.30*	0.0576	1.06 (1.00-1.12)
		GG	0.11	0.09	0.0423	/	0.10	0.10	0.4829	/	0.10	0.10	0.1398	/
		GA	0.42	0.41			0.43	0.42			0.43	0.41		
		AA	0.46	0.50			0.47	0.49			0.47	0.49		
rs6669008	114166561	G<A	0.28*	0.24*	0.0004	1.23 (1.09-1.38)	0.26*	0.25*	0.3158	1.04 (0.97-1.11)	0.26*	0.25*	0.0095	1.08 (1.02-1.15)
		GG	0.08	0.06	0.0029	/	0.06	0.06	0.5702	/	0.07	0.06	0.0345	/
		GA	0.39	0.40			0.40	0.39			0.40	0.38		
		AA	0.53	0.54			0.54	0.55			0.54	0.56		
rs1230647	114253639	T<C	0.37*	0.33*	0.0023	1.19 (1.06-1.32)	0.35*	0.34	0.2010	1.04 (0.98-1.11)	0.36*	0.34*	0.0081	1.08 (1.02-1.14)
		TT	0.14	0.11	0.0077	/	0.13	0.12	0.4119	/	0.13	0.12	0.0239	/
		TC	0.45	0.44			0.45	0.45			0.45	0.45		
		CC	0.41	0.45			0.42	0.43			0.42	0.44		
rs3811021	114356663	G<A	0.22*	0.19*	0.0010	1.24 (1.09-1.41)	0.22*	0.21*	0.1383	1.06 (0.98-1.14)	0.22*	0.21*	0.0033	1.10 (1.03-1.17)
		GG	0.06	0.03	0.0013	/	0.05	0.13	0.2340	/	0.05	0.04	0.0132	/
		GA	0.33	0.31			0.39	0.74			0.34	0.32		
		AA	0.61	0.65			0.56	0.13			0.61	0.63		
rs1746853	114383097	C<A	0.27*	0.24*	0.0149	1.16 (1.03-1.31)	0.26*	0.25*	0.1204	1.06 (0.99-1.13)	0.26*	0.25*	0.0102	1.08 (1.02-1.15)
		CC	0.08	0.06	0.0205	/	0.07	0.07	0.2974	/	0.07	0.06	0.0271	/
		CA	0.37	0.36			0.38	0.37			0.38	0.37		
		AA	0.55	0.58			0.55	0.56			0.55	0.57		
rs2358994	114429461	G<A	0.36*	0.33*	0.0432	1.12 (1.01-1.25)	0.34*	0.33*	0.2892	1.04 (0.97-1.10)	0.35*	0.33*	0.0521	1.06 (1.00-1.12)
		GG	0.13	0.12	0.0776	/	0.12	0.12	0.5684	/	0.12	0.12	0.1292	/
		GA	0.45	0.42			0.44	0.43			0.44	0.43		
		AA	0.42	0.46			0.44	0.45			0.43	0.45		
rs17464525	114443899	A<G	0.18*	0.15*	0.0073	1.21 (1.05-1.39)	0.18*	0.17*	0.2464	1.05 (0.97-1.14)	0.18*	0.17*	0.0191	1.09 (1.01-1.16)
		AA	0.04	0.02	0.0106	/	0.03	0.03	0.1213	/	0.03	0.03	0.0460	/
		AG	0.29	0.27			0.29	0.27			0.29	0.27		
		GG	0.68	0.71			0.68	0.69			0.68	0.70		

Note: SNP: single nucleotide polymorphism, MAF: minor allele frequency, OR: odds ratio for the minor allele. * MAF

Based on the 186 genotyped SNPs in 1,442 GD patients and 1,468 control subjects, the genotype frequencies of 1,227 SNPs in the LD block containing *PTPN22* were obtained by imputation analysis (Figure 1A and Table S1). Interestingly, the SNPs, including the genotyped and the imputed SNPs, located in an LD block of about ~370 kb containing seven genes (*MAGI3, PHTF1, RSBN1, PTPN22, BCL2L15, AP4B1*, and *DCLRE1*) at 1p13.2, were strongly associated with GD in our data. The most significant association signal surrounded *PTPN22* (*P*_GWAS_=0.0007, OR=1.23, 95%CI: 1.09-1.38 for rs6669008), whereas the association signals in the region outside the ~370 kb LD block were relatively weak (Figure 1B; Tables S1 and S2). 

### Replication Study and the Combined Analysis

Base on LD analysis in our GWAS data, nine tagSNPs were able to fully tag (r^2^>0.8) the 255 SNPs with *P*_GWAS_ <0.05 in the ~370 kb LD block containing *PTPN22* (Table S2). Although the P value of the all 255 SNPs are more than 0.000196 (0.05/255), the nine tagSNPs were further selected and genotyped in a cohort of 4,368 GD patients and 4,350 control subjects in the replication study given that the genotypes of some SNPs were obtained by imputation. 

Unexpectedly, our data revealed that all nine tagSNPs were not associated with GD in the replication study (Table 1). Specifically, no significant differences in allele or genotype frequencies were observed between the GD patients and healthy controls (*P*_replicated_ =0.3158 for rs6669008, OR=1.04, 95% CI: 0.97–1.11; Table 1), despite good statistical power (nearly 100%) to detect an effect size of 1.2 (Table 2).

**Table 2:** False positive report probability (FPRP) values for nine replicated SNPs between 5,696 GD patients and 5,767 health individuals.

SNP	Reported *P*-Value	Reported OR	OR	Statistical power under recessive model *	Prior probability					
					0.25	0.1	0.01	0.001	0.0001	0.00001
rs7514649	0.0468	1.07	1.15	0.98	**0.1255**	0.3009	0.8256	0.9795	0.9979	0.9998
			1.20	1.00	**0.1231**	0.2963	0.8224	0.9791	0.9979	0.9998
			1.50	1.00	**0.1230**	0.2962	0.8224	0.9790	0.9979	0.9998
rs12753075	0.4635	1.04	1.15	0.99	**0.5852**	0.8089	0.9790	0.9979	0.9998	1.0000
			1.20	1.00	**0.5819**	0.8068	0.9787	0.9978	0.9998	1.0000
			1.50	1.00	**0.5817**	0.8066	0.9787	0.9978	0.9998	1.0000
rs1217223	0.0576	1.06	1.15	1.00	**0.1475**	0.3416	0.8509	0.9829	0.9983	0.9998
			1.20	1.00	**0.1473**	0.3413	0.8507	0.9829	0.9983	0.9998
			1.50	1.00	**0.1473**	0.3413	0.8507	0.9829	0.9983	0.9998
rs6669008	0.0095	1.08	1.15	0.98	**0.0283**	**0.0803**	0.4898	0.9064	0.9898	0.9990
			1.20	1.00	**0.0276**	**0.0784**	0.4836	0.9043	0.9895	0.9989
			1.50	1.00	**0.0276**	**0.0784**	0.4835	0.9043	0.9895	0.9989
rs1230647	0.0081	1.08	1.15	1.00	**0.0238**	**0.0682**	0.4462	0.8905	0.9879	0.9988
			1.20	1.00	**0.0238**	**0.0681**	0.4455	0.8902	0.9878	0.9988
			1.50	1.00	**0.0238**	**0.0681**	0.4455	0.8902	0.9878	0.9988
rs3811021	0.0033	1.10	1.15	0.92	**0.0106**	**0.0310**	0.2606	0.7805	0.9727	0.9972
			1.20	1.00	**0.0097**	**0.0286**	0.2448	0.7659	0.9704	0.9970
			1.50	1.00	**0.0097**	**0.0285**	0.2442	0.7652	0.9703	0.9969
rs1746853	0.0102	1.08	1.15	0.98	**0.0302**	**0.0854**	0.5067	0.9120	0.9905	0.9990
			1.20	1.00	**0.0296**	**0.0839**	0.5018	0.9104	0.9903	0.9990
			1.50	1.00	**0.0296**	**0.0839**	0.5017	0.9104	0.9903	0.9990
rs2358994	0.0521	1.06	1.15	1.00	**0.1353**	0.3194	0.8377	0.9812	0.9981	0.9998
			1.20	1.00	**0.1352**	0.3192	0.8376	0.9811	0.9981	0.9998
			1.50	1.00	**0.1352**	0.3192	0.8376	0.9811	0.9981	0.9998
rs17464525	0.0191	1.09	1.15	0.95	**0.0569**	**0.1532**	0.6656	0.9526	0.9950	0.9995
			1.20	1.00	**0.0544**	**0.1471**	0.6548	0.9503	0.9948	0.9995
			1.50	1.00	**0.0542**	**0.1468**	0.6543	0.9503	0.9948	0.9995

* Statistical power was the power to detect an odds ratio respectively of 1.12,1.20,1.5 for the homozygotes with the rare genetic variant, with an α level equal to the reported P-value. FPRP values below 0.2 were shown in bold letters.

We further analyzed the LD structure of the nine tagSNPs in case and control subjects based on data from our replication cohort. However, there are no significant differences of the LD structure between GD patients and control subjects, which are also similar with the LD structure of Asia population using the data from 1000-Human-Genome (Table S2, Figure S1). The data suggested that the imputation data in current study are acceptable. Thus, we combined the results of the GWAS and replication stages, and six out of nine tagSNPs in the ~370-kb LD block containing *PTPN22* were found norminal associated with GD, with the most association signal at rs3811021 (*P*_combined_ = 0.0033, OR=1.10, 95% CI: 1.03–1.17;), but no SNPs with *P* value are less than 0.000196 (0.05/255), the threshold of significant in the current study (Table 1). 

### FPRP analysis

 In order to determine whether the combined result showing nominal association between the 6 SNPs and GD was a false positive signal, the FPRP was analyzed[34]. Here, the FPRP value was calculated under an assigned prior probability ranging from 0.00001 to 0.25, using the statistical power to detect an OR of 1.2 and the observed ORs and *P* values. Our case-control study for the nine SNPs in a total sample of 5,696 patients with GD and 5,767 control individuals has more than 99.5% statistical power to detect a SNP with a level equal to its reported *P* value, corresponding to relative risks of 1.2 for GD (Table 2). Notably, the FPRP values of SNP rs3811021 (*P*_combined_ = 0.0033) were below 0.2 just for the prior probability at 0.25 which was just a relatively high prior probability range. However, the values were more than 0.2 if the prior probability was less than 0.25, suggesting that the six SNPs with a week association signal with GD may be caused by false positive reports.

### Polymorphism comparison in the present and previous studies of GD

Until now, most studies of GD mainly focused on investigating the association of functional SNP rs2476601 with GD in Caucasian populations (Table 3)[21,22,36-39]. However, a few studies were carried out in Asian populations to investigate the GD associations of SNPs in *PTPN22* other than rs2476601 (Table 3)[19,28,29,40]. It is worth noting that a study conducted in a United Kingdom Caucasian population (768 GD patients, 768 control subjects) showed no association with GD of any of the 5 tagSNPs that were selected for genotyping in the *PTPN22* region. However, these 5 tagSNPs were in the lower LD with rs2476601 based on 1000 Genomes project data (Table 3)[36]. Ichimura et al. found that one SNP, rs3789604, was significantly associated with GD in 414 patients and 231 control subjects recruited from a Japanese population (*P*= 0.0085, OR = 1.45; Table 3) [28], which was subsequently replicated by Gu et al. in a Chinese population[19]. Based on our imputation data, rs3789604 is associated with GD (*P*_GWAS_=0.001354, Table 3, Table S1). Although this SNP has not been genotyped in our second cohort, one SNP, rs3811021, in the high LD block with rs3789604 (r^2^ = 0.99) was selected and genotyped in the replication study. The allele frequency of rs3811021 did not differ significantly between the 4,368 GD patients and the 4,350 controls in the replication study (*P*=0.138, OR=1.06). Another study performed in a Korean population by Lee et al. reported that rs12730735 was associated with susceptibility to autoimmune thyroid disorders (AITDs) in a total of 212 AITD (84 GD and 128 Hashimoto's thyroiditis) patients and 225 controls, especially with that to Hashimoto's thyroiditis (*P* < 0.01)[40]. However, there was no evidence to support the association of rs12730735 with GD in our GWAS data (*P*_GWAS_ = 0.1857; Table 3, Table S1), and rs12753075, in a high LD block with rs12730735 (r^2^=0.93), was also not associated with GD in the replication study (*P*_replicated_= 0.7315, OR=0.98, Table 1). Although our combined data did not confirmed the association of SNPs at *PTPN22* region with GD, it is necessary to perform a meta-analysis of the SNPs previously reported to be associated with GD, especially in Asian population in future research.

**Table 3:** Comparision of the association of SNPs in *PTPN22* region with GD in the current study with that in previous reports.

SNP	Alleles	Case MAF(%)		Control MAF(%)	Reported P vaule	Study population		Study first author (reference)		P value in our GWAS cohort	r^2^ with the SNP replicated in our cohort
rs3789604	A>C	145/828(17.5)		109/462(23.6)	**0.0085^$^**	Japanese		Ichimura M (40)		**0.0012**	**rs3811021:r^2^=0.91**
	A>C	61/352(17.3)		115/562(20.5)	0.24	Japanese		Ban Y (24)			
	A>C	181/822(22.0)		106/624(17.0)	**0.017**	Chinese		Gu LQ (16)			
rs3811021	T>C	287/1484(19.3)		259/1396(18.6)	0.59	UK Caucasians		Heward JM (35)		**0.0009796** ^#^	
	C>T	83/422(19.7)		95/442(21.5)	0.5	Koreans^*^		Lee HS (39)			
rs1217413	T>C	353/1474(23.9)		338/1484(22.8)	0.451	UK Caucasians		Heward JM (35)		**0.0108**	**rs1230647:r^2^=0.91**
rs2476599	G>A	43/352(12.2)		76/562(13.5)	0.57	Japanese		Ban Y (24)		0.0913	
rs1217388	C>T	157/420(37.4)		148/444(33.3)	0.21	Koreans^*^		Lee HS (39)		**0.0048**	**rs1230647:r^2^=0.97**
rs3789607	T>C	61/828(7.0)		32/462(7.0)	0.7692	Japanese		Ichimura M (40)		0.1464	
	T>C	82/850(9.7)		64/628(10.2)	0.729	Chinese		Gu LQ (16)			
rs1310182	C>T	663/1412(47)		644/1422(45.3)	0.394	UK Caucasians		Heward JM (35)		0.8680	
	T>C	66/352(18.8)		109/562(19.4)	0.81	Japanese		Ban Y (24)			
	T>C	69/424(16.3)		55/444(12.4)	0.1	Koreans^*^		Lee HS (39)			
rs2797415^§^	T>C	140/352(39.8)		228/562(40.6)	0.81	Japanese		Ban Y (24)			
rs1970559	T>C	33/818(4)		28/630(4.4)	0.7	Chinese		Gu LQ (16)		0.10539	
rs2476601^§^	C>T	258/1802(14.3)		174/1666(10.4)	6.26×10-4	UK Caucasians		Smyth D (21)			
	C>T	151/1098(13.8)		67/858(7.8)	3.40×10-5	Caucasian		Velaga MR (20)			
	C>T	112/580(19.3)		76/620(12.3)	0·0008	Polish		Skórka A (36)			
	C>T	0(0)		0(0)	nonpolymorphism	Chinese		Gu LQ (16)			
	C>T	0(0)		0(0)	nonpolymorphism	Japanese		Ban Y (28)			
	C>T	63/342(18.4)		70/400(17.5)	NS	Russian		Zhebrun D (37)			
	C>T	68/396(17.2)		60/396(15.2)	NS	Polish		Jurecka-Lubieniecka B (38)			
	C>T	0(0)		0(0)	nonpolymorphism	Japanese		Ichimura M (40)			
rs12730735	A>G	392/1374(28.5)		391/1390(28.1)	0.815	UK Caucasians		Heward JM (35)		0.1615	
	A>G	39/422(9.2)		22/444(5)	**0.01**	Koreans^*^		Lee HS (39)			
rs12760457	C>T	23/352(6.5)		37/562(6.6)	0.98	Japanese		Ban Y (24)		0.1617	
rs1217419	T>G	117/852(13.7)		92/630(14.6)	0.634	Chinese		Gu LQ (16)		0.88474	
rs2488458	G>A	399/1482(26.9)		362/1436(25.2)	0.292	UK Caucasians		Heward JM (35)		**0.0070**	**rs1230647: r^2^=0.97**
	A>G	298/850(35.1)		220/630(34.9)	0.956	Chinese		Gu LQ (16)			
	A>G	163/424(38.4)		152/444(34.2)	0.19	Koreans^*^		Lee HS (39)			
rs2488457^§^	C>G	339/828(40.9)		202/462(43.7)	0.3318	Japanese		Ichimura M (40)			
	C>G	259/796(32.5)		222/630(35.2)	0.284	Chinese		Gu LQ (16)			

Note: §, The SNPs was not included in our GWAS cohort; *, The study includes 212 AITD (128 Hashimoto’s thyroiditis and 84 Graves’disease) patients, and 225 ethnically matched healthy controls; $, Corrected p-values (Pc)=0.034; #, This SNP did not showed significant difference between GD and controls in our replicaiton stage; NS: non-significant. The P value reported less than 0.05 were highlighted in bold.

## Discussion

Previous studies provided solid evidence for *PTPN22* as a susceptibility gene for GD in Caucasian populations. Notably, the rs2476601 polymorphism was reported monomorphic in Asian populations[19,28-30], which indicates that it may not have a causal role for GD in the Asian population. However, we cannot exclude the *PTPN22* region harboring other susceptibility SNPs for GD in the Chinese Han population. So this phenomenon provided an excellent model to confirm whether GD is a heterogeneous disease in distinct ethnic populations, which may be caused by different major susceptibility genes or different SNP variants in one susceptibility gene. 

Thus, in the current study, nine tagSNPs were selected and genotyped in 4,254 GD and 4,299 control individuals to investigate whether SNPs in the *PTPN22* region were associated with GD in the Chinese Han population. Unexpectedly, all nine tagSNPs were not associated with GD in replicated samples (*P*_replicated_ =0.7315 to 0.1383; Table 1). Although the combined case-control association study still exhibited a nominal association signal (*P*_combined_ = 0.0033, OR=1.10, 95% CI: 1.03–1.17 for rs3811021; Table 1), but the P value of rs3811021 is more than the signifiacant threshold 0.000196 (0.05/255). Moreover, the follow-up FPRP calculation suggested it was most likely a false positive finding (Table 2). We also calculated the power using the CaTS Power Calculator software to replicate the association between the most significant SNP, rs3811021, and GD at the level of *P*<5×10^-8^ and found that the possibility was less than 1% in our current sample size. Quanto software was also used to estimate the sample size needed in the association between rs3811021 and GD to reach the GWAS significance level (*P* < 5×10^-8^) (http://hydra.usc.edu/gxe). It required nearly 26,500 cases and 26,500 controls to achieve this level of significance, which is too large to fulfill in the current stage. The 1000 Genomes Project data also indicated the quite different allelic frequencies of SNPs in the *PTPN22* LD block between different ethnic populations (Table S3), which further demonstrated that *PTPN22* may not associated with GD in the Chinese Han population despite the evidence that *PTPN22* is a susceptibility gene for GD in European Caucasian populations. 

The present study suggests that common SNPs from the *PTPN22* region 1p13.2 were not associated with GD, which provides more solid evidence to assert that *PTPN22* is an ethnicity-specific GD susceptibility gene in Caucasian populations but not in Chinese Han populations. Alhtough the SNP density and sample size in our current study are large enough to provide convincing evidence that *PTPN22* was not associated with GD in Chinese Han population, the target resequencing for the *PTPN22* region in GD from Chinese Han population will be required in the further study, given that the genotypes of some SNPs were obtained by imputation. 

To our knowledge, most of the association studies about *PTPN22* and immune-related disease focused only on rs2476601 in relatively small sample sizes. More recently, several GWAS studies using larger sample sizes also indicated that rs2476601 was strongly associated with some autoimmune diseases, such as T1D , RA, and Crohn's disease, especially T1D, with a P value up to 2×10^-111^ (OR=2.0)[41]. These data strongly support the hypothesis that *PTPN22* is a major susceptibility gene for autoimmune disease in Caucasian populations. In our current study, we conducted a comprehensive refining association analysis of the *PTPN22* region in relatively large GD cohort, allowing us to have good power (nearly 100%) to detect the previously reported association. However, we failed to find any association of *PTPN22* with GD in the Chinese Han population. Moreover, no associations of SNPs in *PTPN22* region with autoimmune diseases were found in Asian populations by searching GWAS data from UCSC website. 

Our results revealed that the SNPs in *PTPN22* were not associated with GD in Chinese Han population. However, it is should be considered that one limitation in the current study is the population stratification in our replication cohort might be influence the conclusion. Although our and other previous researches [16][42,43] did not found significant population stratification in Chinese Han population, it would be much more reasonable to elucidate our negative association results after the population stratification analysis.

In conclusion, we provided the most convincing evidence that *PTPN22* was not associated with GD in Chinese Han population and different susceptible genes were responsible for GD in different ethnics.

## Supporting Information

Figure S1 **Linkage disequilibrium plots of the nine tagSNPs in GD patients (**A**), controls subjects (**B**) of replication stage and healthy individuals (**C**) from 1000-Human-Genome Asia population.** The color of each SNP spot reflects its r2, with the top typed SNP (large red diamond) within each association locus changing from black to white.
(JPG)

Table S1 **Note: SNP: single-nucleotide polymorphism, MAF: minor allele frequency, OR: odds ratio for the minor allele.** SNPs with P-values less than 0.05 are in bold letters.
(XLS)

Table S2 **Note: SNP: single-nucleotide polymorphism, MAF: minor allele frequency, OR: odds ratio for the minor allele, r2: r square.**
(XLS)

Table S3 **Note: SNP: single-nucleotide polymorphism, MAF: minor allele frequency, OR: odds ratio for the minor allele.** ASN:All East Asian individuals from phase 1 of the 1000 Genomes Project (CHB, JPT, CHS), EUR: All European individuals from phase 1 of the 1000 Genomes Project (CEU, TSI, FIN, GBR, IBS), AFR:All African individuals from phase 1 of the 1000 Genomes Project (YRI, LWK, ASW), AMR: All American individuals from phase 1 of the 1000 Genomes Project.
(XLS)
