# Supplementary figures and images for: Genetic Heterogeneity of Susceptibility Gene in Different Ethnic Populations: Refining Association Study of PTPN22 for Graves’ Disease in a Chinese Han Population
Source: PLoS One. 2013 Dec 30;8(12):e84514. doi: 10.1371/journal.pone.0084514 (PMC3875558; doi:10.1371/journal.pone.0084514)

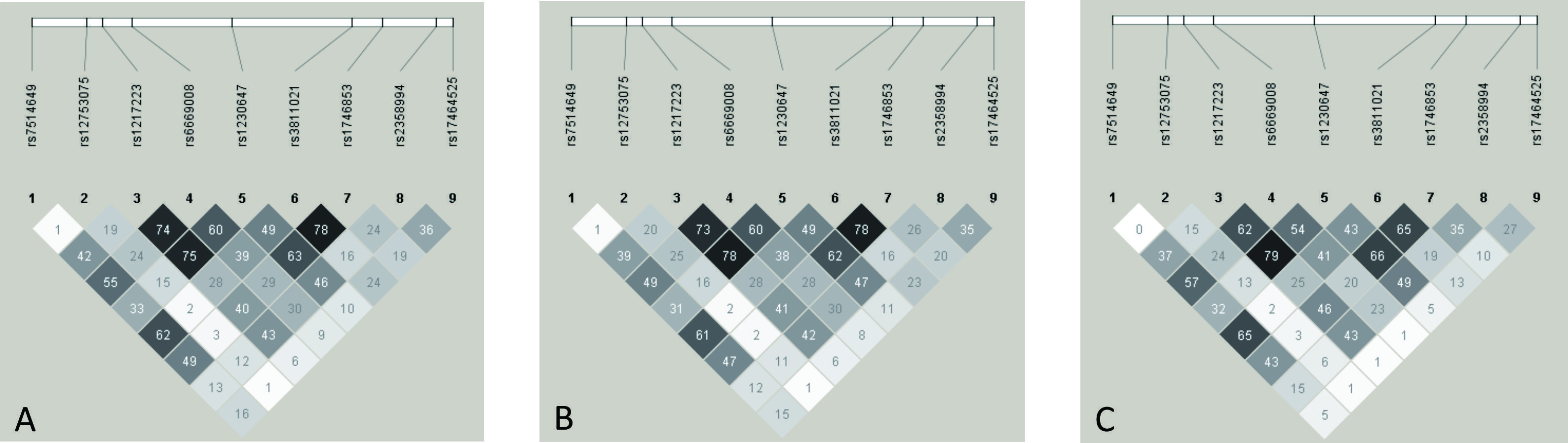

Supplement: Figure S1 — Linkage disequilibrium plots of the nine tagSNPs in GD patients (A), controls subjects (B) of replication stage and healthy individuals (C) from 1000-Human-Genome Asia population. The color of each SNP spot reflects its r2, with the top typed SNP (large red diamond) within each association locus changing from black to white. (JPG) [file pone.0084514.s001.jpg]
